# Treatment of Major Depressive Disorder with Autism Spectrum Disorder by Acceptance and Commitment Therapy Matrix

**DOI:** 10.1155/2021/5511232

**Published:** 2021-04-07

**Authors:** Takafumi Watanabe

**Affiliations:** Department of Psychiatry, Nagoya City University Graduate School of Medical Sciences, 1, Kawasumi, Mizuho-Cho, Mizuho-Ku, Nagoya City, Aichi 467-8601, Japan

## Abstract

Autism spectrum disorder (ASD) is known to increase a variety of psychiatric disorders, including major depressive disorder (MDD). Acceptance and commitment therapy (ACT) has been reported to be an effective psychotherapy for MDD. However, this is not the case with ASD. In this case study, based on the hypothesis that it is effective in treating MDD with ASD, we adapted the ACT Matrix, a tool for visualizing an individual's experiences in the context of ACT, and administered 15 sessions to a woman with MDD with ASD following the onset of photo dermatosis. By the end of the final session, there was an increase in value-based behaviors and significant changes in depressive symptoms as well as significant changes in process measures such as experience avoidance, mindfulness, and progress toward a valued life. Six months after the end of the session, the improvement in symptoms was even stronger and the process measure of obstruction to a valued life was significantly improved. The ACT Matrix may thus ameliorate MDD in patients with ASD.

## 1. Introduction

Autism spectrum disorder (ASD) is characterized by impairments in social interaction, communication, and behavioral flexibility and is found in about 1% of the population [1]. Even the lowest severity of ASD increases the risk for other types of mental health problems. In a recent meta-analysis, the lifetime prevalence of adults with ASD was 11% for depressive disorder [2].

**Figure 1:**
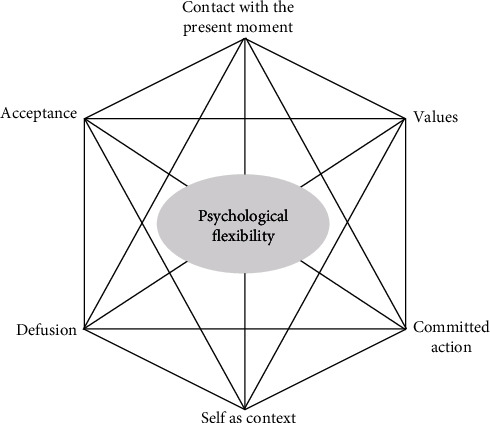
Image of hexaflex. Six interrelated core ACT processes produce psychological flexibility. These processes are (1) contact with the present moment, (2) defusion, (3) acceptance, (4) self as context, (5) values, and (6) committed action.

Acceptance and commitment therapy (ACT) is derived from the modern theory of cognition and language and is classified as a third-wave psychological treatment, in which some cognitive behavioral therapy (CBT) concepts have been changed [3]. The main premise underlying ACT is that humans experience disturbing thoughts, emotions, and feelings when they move toward individual values and that their attempts to change or to remove these experiences are mostly ineffective, which sometimes increases these disturbances and ultimately leads to avoidance or moving away from individual values. ACT focuses on making contact with the present moment and changing or persisting in behavior to reach value-consistent goals, or “psychological flexibility.” Psychological flexibility is established through six core ACT processes, collectively known as the hexaflex (Figure 1). The effectiveness of ACT has been reported for MDD [4, 5]. However, in the treatment of MDD with ASD, the effectiveness of any psychotherapies including ACT has not been well examined.

The ACT Matrix (matrix) is a visual tool that helps individuals categorize their experiences along two dimensions to target psychological flexibility in the moment-to-moment experience. The matrix is a diagram of noticing that is composed of two bisecting lines. The vertical line divides an area into two parts, one of which includes the aspect of our experience coming through our five senses: vision, hearing, smell, taste, and touch, and another, which includes the aspect of our experience arising from our mental activity or interoceptive abilities. The horizontal line divides each half into two parts, one of which contains actions aimed at moving away from unwanted experiences, and the other contains actions aimed at moving toward someone or something important. The therapist invites a client to sort and point out their experiences into the quadrants formed by the two bisecting lines. The client thereby acquires psychological flexibility by noticing the difference between the five senses and mental experience and noticing the difference between moving toward who or what is important and moving away from unwanted inner experiences [6]. Although the matrix is a great way to practice perspective taking skills visually, by just pointing out the differences, and is speculated to be a useful tool for MDD with ASD, there have been no reports as yet. The following case study describes how the ACT Matrix was applied to a Japanese female whose diagnosis was MDD with ASD in enabling her valued behaviors.

## 2. Case Presentation

### 2.1. History

The information of the client has been modified to ensure anonymity. The patient was a 42-year-old unmarried Japanese woman who lived with her father, mother, and elder sister. Ever since she had been a little girl, she was not so good at imagining and communicating people's feelings that she avoided speaking in public. Her academic performance in junior high school was unsatisfactory, so she dropped out of high school. After working in a clothing store and studying in a sewing school, she opened a clothing shop with her sister and a boyfriend in her early 20s. She closed it because she broke up with her boyfriend and started working part-time at a bar at night, but gradually became depressed. She has been attending a psychiatric clinic for a diagnosis of MDD since her early 30s, and began to work in her sister's clothing store after partial remission of MDD, where she designed, sewed, and sold clothes. Three months before being referred to the psychiatric outpatient clinic at the facility where the author worked, she noticed that her face, neck, and shoulders burned and was diagnosed with rosacea, a photo-aggravated dermatosis by a dermatologist. She began to avoid going out, dealing with the customers, and talking with her friends due to erythrophobia. She felt depressed and blamed herself, not helping at her sister's job. Her dermatologist introduced her to the psychiatric unit for her depression and the author examined her.

The therapist was a 48-year-old man with an MD and a license to practice as a psychiatric specialist and clinical psychologist in Japan. He was familiar with CBT and ACT and was certified as a CBT therapist at the Center for the Development of Cognitive Behavioral Therapy Training in Japan. He has been an ACT therapist in the Association for Contextual Behavioral Science and a supervisory doctor of psychiatry certified by the Japanese Society of Psychiatry and Neurology.

### 2.2. Case Formulation

The patient expressed that her difficulties of social communications that had existed since elementary school age worsened due to increased erythrophobia caused by rosacea. She suffered from facial pain and itching, feared that people would give her negative feedback about her blush. She had spent much time lying on the sofa at home. She reported symptoms of MDD, such as sustained depressive mood, markedly diminished interest, loss of energy, appetite loss, sleep disturbance, and feelings of worthlessness, but not suicidal ideation.

To confirm her autistic traits, the Autism-Spectrum Quotient Japanese-21 (AQ-J-21) [7] that is a 21-item questionnaire (4 for social skill; 2, attention switching; 3, attention to detail; 8, communication; and 4, imagination), was conducted. AQ-J-21 is a short form of the AQ-J originating from AQ, which was developed for the ASD screening [8]. AQ-J-21 has high internal consistency (Cronbach's *α* = 0.71, *n* = 240), and its sensitivity, specificity, and positive and negative predictive values were 0.92, 0.81, 0.35, and 0.99, respectively, at a cut-off of 12 for ASD diagnosis. AQ-J-21 showed the following scores: social skills, 1 point out of 4; communication, 3 out of 8; attention to detail, 3 out of 3; attention switch, 2 out of 2; imagination, 3 out of 4; and total score, 12 out of 21. It was expected that she would pay exceptional attention to detail and would not switch attention adequately or understand the other person's perspective.

The author diagnosed her with MDD and ASD according to *Diagnostic and Statistical Manual of Mental Disorders* (5th ed.; *DSM-5* American Psychiatric Association (APA), 2013), and treated her primarily with pharmacotherapy in a general psychiatric outpatient practice. Although 10 mg of escitalopram and 0.25 mg of brotizolam had been prescribed for 4 weeks, her symptoms of MDD had worsened. She had spent much time in her room ruminating about the past and the future, had become irritable toward her family, and had not seen her friends. The author decided to set aside time outside of the general psychiatric outpatient clinic to conduct a more detailed psychobehavioral assessment.

She had several psychological inflexibilities from an ACT perspective. She strongly avoided situations that induced aversive body sensations, such as doing exercise and meeting with others not only outside but also inside the house, although the dermatologist did not forbid her from doing so. She was fused with various thoughts, such as “Am I the only one who has had such a bad skin disease, although I had originally been easy to blush?” and “I would have never been able to go out alone again.” It was surmised that she had many non-functional behaviors (e.g., always putting on a mask everywhere including a consultation room, speaking with her family in a nasty tone, and ceasing communication with her friends). It was expected that ACT would be useful for non-functional behaviors associated with the experiential avoidance and the fusion of thinking, but because of the comorbidity of ASD, it was decided to use ACT matrix.

### 2.3. Assessment

The Quick Inventory of Depressive Symptomatology Self Report-Japanese (QIDS-SR-J) [9] is a 16-item questionnaire that contains nine symptom domains of MDD and rates the severity of MDD, originating from the QIDS-SR [10]. QIDS-SR-J has high internal consistency (Cronbach's *α* = 0.86, *n* = 29) in Japan. The total QIDS-SR-J score ranges from 0 to 27, where a cutoff value of ≤6 represents remission.

The Cognitive Fusion Questionnaire-7 Japanese (CFQ-7-J) [11] is a measure of cognitive fusion composed of seven items originating from CFQ-7 [12]. Cognitive fusion is the behavioral process in which cognitive influence dominates over direct experiential influence without distancing from thought. CFQ-7-J has high internal consistency (Cronbach's *α* = 0.91), and the mean score was 27.8 (SD = 8.76, *n* = 345) in a nonclinical sample of Japanese university students.

The Mindful Attention Awareness Scale-Japanese (MAAS-J) [13] is a 15-item measure of mindfulness designed to measure present moment attention and awareness, originating from the MAAS [14]. MAAS-J has high internal consistency (Cronbach's *α* = 0.93) and a mean score of 66.92 (SD = 11.52, *n* = 377) in a nonclinical sample of Japanese community adults.

The Valuing Questionnaire-Japanese (VQ-J) [15] is a 10-item measure of valued living for which a two-factor solution (factor 1 = progress (in valued living); factor 2 = obstruction (to valued living)) was supported by exploratory and confirmatory factor analysis in a nonclinical sample of Japanese university students, originating from VQ [16]. VQ-J obstruction had high internal reliability (Cronbach's *α* = 0.50) and the mean score was 16.6 (SD = 4.55, *n* = 262) and VQ-J progress had high internal reliability (Cronbach's *α* = 0.82), and the mean score was 15.9 (SD = 5.32, *n* = 261) in a nonclinical sample of Japanese university students.

The QIDS-SR-J as a symptom measure and the CFQ-7-J, MAAS-J, and VQ-J as process measures related to psychological flexibility were monitored at intake, in every session, and at 6 months posttreatment.

### 2.4. Procedure

The intervention protocol was based on Polk et al.'s (2016) step-by-step approach to using the ACT Matrix model. Idiosyncratic adjustments were made to the protocol length in light of her experience and progress made during session. Twelve to 16 sessions were predetermined for an hour each time at intervals of 1 or 2 weeks. Her psychotropic medication (escitalopram; 10 mg daily, and brotizolam; 0.25 mg daily) did not change during the intervention. She was adherent to the medication throughout the treatment.

She was asked to choose clinically relevant target behaviors (CRBs) consistent with her values and to record them in her daily activity log after the intake for daily self-monitoring. CRBs for the time being were to go out and to get up earlier than 11 am.

She was introduced to the matrix in the first session. She was asked to fill in the lower right quadrant of the matrix with the people and things that were important to her, and to fill in the lower left quadrant with the barriers that emerged in her internal experience. She was further explained that the upper regions of the matrix would indicate how her behavior would appear to others, and she was asked to fill in the upper right quadrant with behaviors toward people and things that were important to her, and to fill in the upper left quadrant with behaviors that would keep her away from unpleasant experiences. To exercise noticing herself not just thinking and feeling, but also perceiving them with five senses, the therapist introduced a mindful breathing exercise, which took about 5 minutes. She was encouraged to perform a mindful breathing exercise at home between sessions. Furthermore, once every day, she was asked to reply to simple questions sent by e-mail as follows: “Right now, are you moving toward or away from value?,” and “If you are moving toward value, how hard was it to get close to value, very hard, a little hard, or not hard?” The forms of these questions were automatically e-mailed every day.

### 2.5. Course of Treatment

The patient practiced a mindful breathing exercise 1 to 3 times daily, although she was not good at expressing her sentiments concerning the exercise very well in the beginning. The therapist reinforced the continued practice of mindful breathing by supporting her to notice something in the matrix. For example, she noticed her inner experiences by writing in the right lower quadrant of the matrix, “me,” “my sister,” “walking,” and “health,” and by writing in the left lower quadrant of the matrix, “depression and fatigue,” “image of red face,” “hot, facial pain, and itching,” and “I don't want get up because I would recall my illness many times.” Next, she filled out “ignoring friends' e-mails,” “sitting speechless,” and “taking it out on my cat” in the left upper quadrant of matrix, and “walking outside,” “playing with my cat,” and “shopping” in the right upper quadrant of matrix. The therapist encouraged her to perform a functional assessment of her away moves. That is, in the left upper quadrant she assessed the short-term and long-term effectiveness of her behaviors to avoid or control unwanted experiences on a scale of 1–10. She found that the long-term effectiveness of all away moves chosen by her was much lower than the short-term effectiveness of those moves. She also assessed the importance of away moves on a scale of 1–10, and found that almost all scores of away moves were 1. The therapist told her that when she is moving toward someone or something important in her daily life, the obstacles inside of her that come up are metaphorically like “baited hooks,” and she could observe whether she bites and struggles when she notices the hooks, or whether she just lets them be. Although it was difficult for her to speak the difference of between moving toward someone or something important and moving away from inner obstacles in a verbal way, the therapist encouraged her to just notice the difference using the matrix in a less verbal way.

**Figure 2:**
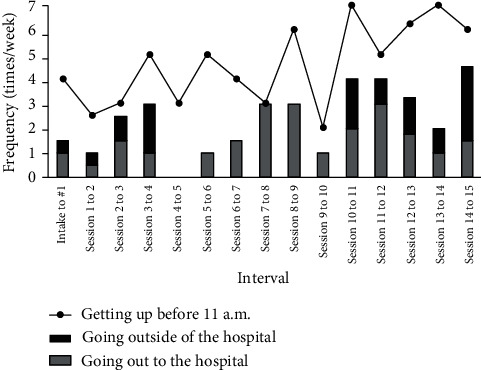
The frequency of clinically relevant target behaviors consistent with the patient's values from intake to the end of sessions.

Although the mean days per week when she went out both for hospital visits and for other purposes gradually increased from session 1 to session 4, they disappeared from session 5 to session 10 (Figure 2), because she focused on daily activities at home including the exercise to discriminate between an away and a toward moves in the present moment. As she accustomed herself to living a mindful life at home and the number of days per week when she got up earlier than 11 am increased, she came to be able to do her housework to help her family and gradually to go out alone and to work at the clothing store to help her sister. The increase in days per week when she went out for other reasons resumed and persisted from session 11. After the intervention was completed in session 15, she has had a short checkup every 4 to 6 weeks in a general psychiatric outpatient practice. She has worked manufacturing apparel with her sister, helps her mother with domestic work, and has resumed contact with her friends.

She responded to the e-mail questions every day. Not once did she answer “moving away from value”, but all the way up to session 6 she answered “very hard”. She replied “a little hard” more often than “very hard” from session 7 and almost always replied “a little hard” from session 10. By decreasing the difficulty of engaging in a toward move, it was assumed that she had been able to increase her behavioral repertoire not only at home but also outside since session 11.

**Figure 3:**
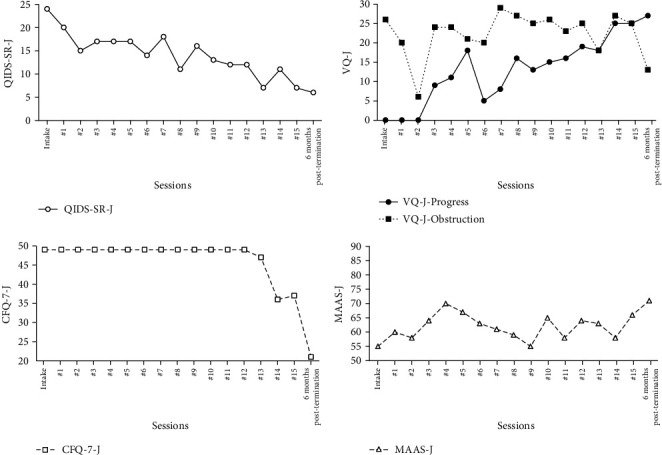
Symptom and process measures from the intake to 6-month posttermination of sessions. QIDS-SR-J = Quick Inventory of Depressive Symptomatology Self Report-Japanese; AAQ-II-J = second version of the Acceptance and Action Questionnaire-Japanese; CFQ-7-J = Cognitive Fusion Questionnaire-7 Japanese; MAAS-J = Mindful Attention Awareness Scale-Japanese; VQ-J = Valuing Questionnaire-Japanese.

The changes in symptom and process measures are presented in Figure 3. The QIDS-SR-J score, which rates depressive symptoms, decreased from 24 points to 7 points at the end of sessions, which is almost subclinical, consistent with the increase in her self-monitored behaviors. The improvement in depressive symptoms persisted even six months after termination of the intervention. A reliable change index (RCI) [17] was computed to evaluate the reliability and statistical significance of the difference between the baseline (intake) and/or termination (session15) and/or follow-up 6 months posttermination scores in the standard measures (Table 1). Statistical significance was computed by dividing the difference between baseline and posttreatment scores on a measure by the standard error of the difference. A score of –1.96 to 1.96 on RCI was within the 96% confidence interval, where a score exceeding the *z*-score for the 97th percentile (–1.96 or 1.96) indicates statistical significance (*p* < 0.05). The change in QIDS-SR-J scores from the intake session to the end of sessions revealed a statistically significant reduction in depression symptoms (RCI = –6.56). The reduction of QIDS-SR-J scores also were significant between the baseline and the follow-up 6 months after termination (RCI = −6.94).

Her score for VQ-J progress score increased nearly continuously from session 3 except for session 6, when she noticed that she had not resumed work yet. The RCI score for VQ-J progress between the baseline and the end of sessions was significantly increased (RCI = 9.16), and persisted until 6 months after termination (RCI = 9.89).

The VQ-J obstruction score transiently decreased from baseline to session 2, but ascended again from session 3, probably because she began progressing toward value and noticing her inner experience. The score of VQ-J obstruction gradually decreased again from session 8. However, the RCI score for VQ-J obstruction between the baseline and the end of sessions was not significant (RCI = –0.18). This suggests that she still struggled against her “hooks” even though she had progressed more than ever in valuing living at the end of sessions. Importantly, the RCI score for VQ-J obstruction was significantly reduced between the baseline and follow-up 6 months posttermination (RCI = −2.44).

The findings that her CFQ-7-J scores had remained relatively high before session 13 and that her MAAS-J scores had remained relatively low before the end of sessions might indicate that her skill of mindfulness had finally borne fruit at the end of intervention. RCI scores for the CFQ-7-J between the baseline and the end of session significantly decreased (RCI = −3.23), and the RCI score for MAAS-J significantly increased (RCI = 2.55). Again, RCI scores for the CFQ-7-J between the baseline and follow-up 6 months posttermination significantly decreased (RCI = −7.53), and the RCI score for MAAS-J significantly increased (RCI = 3.71).

## 3. Discussion

This case study shows that the application of the ACT matrix may be effective for MDD with ASD. Although application of ACT for her seemed challenging because of comorbid ASD, her depressive symptom had continued to improve further at 6 months after the end of therapy, and the range of behaviors that seemed to align with her values also expanded.

As the exercises using the matrix diagrams are repeated practices of “just discriminating” whether one is moving toward someone or something important or is moving away from unwanted internal experiences and from the external perspective of the five senses or from the internal perspective of the mind, it is not necessary to verbalize thoughts, feelings, impulses, and images in detail [6]. Rather, it is better to continue the practice of discrimination. In fact, in this case, continued practice of discrimination might have led to subsequent improvement. Future research is needed to determine which characteristics are useful for clients (e.g., visually dominant or auditory significant, and concomitant ADHD symptoms).

She suffered from rosacea as the last comorbidity in addition to MDD and ASD. Photosensitivity has a negative psychological impact [18–20]. She learned to cope with the effects on her of her somatic symptoms, such as facial pain and itching, during ACT sessions. There has already been a growing body of promising work for ACT to chronic physical disease and chronic pain [21, 22]. The present study also showed that ACT may be effective for MDD associated with physical symptoms of the skin, such as photosensitivity.

Notably, her symptoms had continued to improve further at 6 months after the end of therapy. The range of behaviors that seemed to align with her values also expanded. On the process measures, the RCIs of CFQ-7-J and VQ-J obstruct had changed significantly at 6 months posttherapy compared to the end of therapy, in addition to the CFQ-7-J, MAAS-J, and VQ-J progress, which had already changed significantly by the end of treatment. This may indicate that her symptoms improved as she learned more psychological flexibility, such as defusion and acceptance, as she continued with the exercises for 6 months.

As a recommendation to clinicians, the ACT matrix does not require verbalization of thoughts, feelings, impulses, or images; it is better to train psychological flexibility through repeated practice of “just discriminating” with the matrix. In addition to symptom and process measures, it is also important to apply behavioral indicators in line with the client's values whenever possible, and to use the behaviors as indicators of improvement. While it can be difficult to set behavioral indicators at the beginning of sessions, in this case we were able to directly identify improvements in her life using CRBs consistent with her values as indicators, not just a depressive symptom. The use of e-mail reminders is recommended because it can be an exercise in mindful awareness of the internal sensory orientation “in the moment” (i.e., toward a value or away from unwanted internal experiences), as well as a tool for the therapist to assess the client's situation in real time.

## Figures and Tables

**Table 1:** RCIs.

	Baseline (intake)	Termination (session 15)	6 months posttermination	RCI		
				Intake vs. session 15	Intake vs. 6 months posttermination	Session 15 vs. 6 months posttermination
QIDS-SR-J	24	7	6	−6.56^a^	−6.94^a^	−0.39
CFQ-7-J	49	37	21	−3.23^a^	−7.53^a^	−4.31^a^
MAAS-J	55	66	71	2.55^a^	3.71^a^	1.16
VQ-J progress	0	25	27	9.16^a^	9.89^a^	0.73
VQ-J obstruction	26	25	13	−0.19	−2.44^a^	−2.26^a^

RCI = reliable change index; QIDS-SR-J = Quick Inventory of Depressive Symptomatology Self Report-Japanese; CFQ-7-J = Cognitive Fusion Questionnaire-7 Japanese; MAAS-J = Mindful Attention Awareness Scale-Japanese; VQ-J = Valuing Questionnaire-Japanese. ^a^Clinically significant (>1.96).

## Data Availability

The data used to underpin the findings of this study are included within the paper.
